# Crenactin from *Pyrobaculum calidifontis* is closely related to actin in structure and forms steep helical filaments

**DOI:** 10.1016/j.febslet.2014.01.029

**Published:** 2014-03-03

**Authors:** Thierry Izoré, Ramona Duman, Danguole Kureisaite-Ciziene, Jan Löwe

**Affiliations:** MRC Laboratory of Molecular Biology, Structural Studies Division, Francis Crick Avenue, Cambridge CB2 0QH, United Kingdom

**Keywords:** Actin, MreB, Crenarchaea, Bacterial cytoskeleton, Helical filament, Cytomotive filament

## Abstract

Polymerising proteins of the actin family are nearly ubiquitous. Crenactins, restricted to Crenarchaea, are more closely related to actin than bacterial MreB. Crenactins occur in gene clusters hinting at an unknown, but conserved function. We solved the crystal structure of crenactin at 3.2 Å resolution. The protein crystallises as a continuous right-handed helix with 8 subunits per complete turn, spanning 419 Å. The structure of crenactin shows several loops that are longer than in actin, but overall, crenactin is closely related to eukaryotic actin, with an RMSD of 1.6 Å. Crenactin filaments imaged by electron microscopy showed polymers with very similar helical parameters.

## Highlights

• Crystal structure of crenactin solved in polymeric form at 3.2 Å resolution.
• Crenactin is closely related to eukaryotic actin, RMSD 1.6 Å.
• Crenactin forms right-handed filaments with 8 subunits per complete turn.
• Rare crystal structure of helical filament of actin-like protein.

## 1 Introduction

Actin- and tubulin-like proteins are nearly ubiquitous in all three kingdoms of life [1], [2]. Actins’ primary function seems to be regulated polymerisation into cytomotive filaments and for this they contain an ATPase that is sensitive to the polymerisation state [3]. In eukaryotic cells, actins fulfil a plethora of different functions, ranging from cell shape and transport to force generation in muscle and in cytokinesis. Actin-like proteins outside eukaryotes were first discovered using structure-lead sequence database searches [4]: MreB forms antiparallel, straight double filaments, it is present in most non-round bacteria and is involved in the maintenance of cell shape [5], [6]. FtsA, despite having modified domain architecture, polymerises into canonical actin protofilaments and participates in cell division (cytokinesis) together with the bacterial tubulin homologue FtsZ [7]. ParM (StbA) forms left-handed double helical filaments and is involved in low-copy number plasmid segregation, forming mitosis-like spindles that push plasmids to the poles before cell separation [8].

Crenactins were recently discovered following genome sequencing of Crenarchaea [9]. They are more closely related to actins than MreB, although sequence identity is still very low at around 20%. The exact biological function of crenactins is currently unknown, however they were found to be located in functional clusters with other genes, including SMC proteins (Structural Maintenance of Chromosomes), highlighting possible roles in DNA dynamics, for example [9].

The discovery of actin-like proteins other than MreB in Crenarchaea is interesting because of another aspect: it has been postulated that Crenarchaea may have something to do with the cell(s) that took on bacterial endosymbionts to form the eukaryotic lineage and it has recently been proposed that eukaryotes should be regarded as a branch of the Crenarchaea, leading to only two kingdoms of life, instead of three [10]. This would make crenactin the primordial actin; crenactin and MreB would have diverged long before that.

Here we show the crystal structure of crenactin from *Pyrobaculum calidifontis* at 3.2 Å resolution. At 1.6 Å RMSD to yeast actin it is much more related to actin than to MreB. The crystals contain steep helical filaments with 8 subunits per 419 Å complete turn, that are very similar to the crenactin filaments that we observed by electron microscopy.

## 2 Materials and methods

### 2.1 Cloning of crenactin

The crenactin gene from *P. calidifontis* (NCBI YP_001056518.1) was codon-optimised for expression in *Escherichia coli* as supplied by GenScript in a pUC57 vector and used as a template for PCR amplification. The product was subsequently cloned into vector pOPINS (HIS6-SUMO-POI) [11] using ‘In-Fusion Advantage’, according to the protocol in the manufacturer’s instructions (Clontech). The resulting construct encoded a *Pc* crenactin protein N-terminally tagged with a 6-histidine tag followed by a SUMO tag. The polymerisation-deficient mutation V339K was introduced using the QuickChange protocol.

### 2.2 Expression and purification of SUMO protease SENP1

*E. coli* C41(DE3) cells were transformed by electroporation with a pGEX vector containing a GST-tagged C-terminal protease domain of human SUMO protease SENP1. Cells were incubated over night at 37 °C on six agar plates containing 100 μg/ml ampicillin. Cells were harvested directly from the plates to inoculate 12 L of 2×TY media containing 100 μg/ml ampicillin. The culture was first incubated at 37 °C for two hours until OD_600_ reached 0.4, then for 1 h at 18 °C, before protein expression was induced by addition of IPTG to a final concentration of 1 mM and a further incubation at 18 °C for 7 h. The cells were subsequently pelleted by centrifugation, resuspended in 50 mM Tris/HCl, 150 mM NaCl, 1 mM EDTA, 2 mM DTT, pH 8.5, supplemented with small amounts of DNase I and RNase A (Sigma) and protease inhibitors (Roche). Cells were lysed with a Constant Systems cell disruptor at 20 kPSI. The lysate was clarified by ultra centrifugation and incubated with GST beads at 4 °C for 2 h. Beads were extensively washed with 50 mM Tris/HCl, 150 mM NaCl, 1 mM EDTA, 2 mM DTT, pH 8.5 and SUMO protease was eluted by the addition of 10 mM reduced glutathione (Sigma) to the washing buffer. The eluted protein was further purified using a size exclusion column (Sephacryl S-200 16/60, GE Healthcare) pre-equilibrated in 50 mM Tris/HCl, 150 mM NaCl, 2 mM DTT, 1 mM sodium azide, pH 8.5. Fractions containing the protein were pooled, concentrated and flash frozen in liquid nitrogen.

### 2.3 Expression and purification of *P. calidifontis* crenactin

*E. coli* BL21(AI) cells were transformed by electroporation with the pOPINS vector containing the crenactin insert and incubated over night at 37 °C on agar plates containing 50 μg/ml kanamycin. All cells from six such plates were harvested to inoculate 12 L of 2×TY media containing 50 μg/ml kanamycin. The culture was grown at 37 °C until OD_600_ reached 1.00, at which point protein expression was induced for 5–6 h at 37 °C, by addition of arabinose to a final concentration of 0.2%. The cells were pelleted by centrifugation and frozen. The pellet from a 12 l culture was resuspended in 150 ml of buffer A (50 mM Tris/HCl, 50 mM NaCl, pH 8.5), in which 4 EDTA-free protease inhibitor tablets (Roche) had been dissolved. Cells were lysed using a Constant Systems cell disruptor at 20 kPSI. The cell lysate was clarified by centrifugation for 40 min at 42 000 rpm in a Beckman Ti45 rotor. The lysate supernatant was then loaded at 1 ml/min onto a 5 ml HisTrap column (GE Healthcare), pre-equilibrated in Buffer A. The protein was eluted using Buffer A containing 100 mM of imidazole, and concentrated to a final volume of 5 ml, using a Centriprep concentrator (Millipore) with a 10 kDa cut off. Protein concentration was measured using a spectrophotometer and purified SUMO protease SENP1 was added to a final mass ratio of 1:30 of protease to crenactin. The incubation with SUMO protease was performed over night at 4 °C, while dialysing the reaction against Buffer B (25 mM Tris/HCl, 100 mM NaCl, 2 mM DTT and 1 mM EDTA, pH 8.5). In order to remove the SUMO protease, the mixture was subsequently incubated, for 30 min at 4 °C, with a small amount of glutathione-sepharose (GE Healthcare), previously washed in Buffer B. Following centrifugation to remove the resin, the protein solution was loaded onto a Sephacryl S200 16/60 size exclusion column (GE Healthcare), pre-equilibrated in Buffer B. The fractions containing crenactin were checked by SDS–PAGE and then concentrated to 3–8 mg/ml in another Centriprep.

### 2.4 Crystallisation, data collection and structure determination

Crystallisation conditions were found using our in house nanolitre crystallisation facility and crenactin at concentrations around 2–5 mg/ml [12]. Crystals of crenactin were grown by the vapour diffusion method, equilibrating drops of 100 nl reservoir plus 100 nl protein solution against reservoir containing 100 mM phosphate/citrate pH 4.2, 200 mM lithium sulfate and 20% (w/v) PEG 1000. Rod-shaped crystals appeared over 10–20 days. Crystals were cryo-protected by swiftly passing the harvested crystal through a drop of reservoir solution containing in addition 25% glycerol, before flash freezing in liquid nitrogen. Datasets were collected at Diamond Light Source (Harwell, UK) on beamlines I02 and I04-1 on Pilatus detectors and indexed and integrated with XDS [13]. Data reduction was performed with SCALA [14].

Molecular replacement with PHASER 2.5.5 [15] produced a weak hit with 2 molecules of yeast actin (PDB 1YVN) arranged in a helix, indicating a correct solution. The resulting electron densities were unbuildable so multi subunit and multi-crystal averaging in DMMULTI [14] was employed using 4 monomers in datasets Natives 1 and 2 (Table 1), that are highly non-isomorphous but can both be solved by molecular replacement. The resulting phases were greatly improved and enabled REFMAC [16] to reduce the *R*-factors significantly when a model was used that had the sequence of the search model changed to crenactin, based on a multiple sequence alignment. The combined phases after the REFMAC run were then used to let BUCCANEER [17] automatically build the crenactin structure. Several rounds of BUCCANEER, PHASER, DMMULTI and REFMAC were sufficient to arrive at an essentially complete model with *R*-factors of 0.29/0.33 for *R* and *R*-free, respectively. The model was polished using rounds of manual model building in COOT [18] and MAIN [19] and refinement with REFMAC and one molecule of ADP was located and added in each crenactin subunit. The final 2FOFC electron density is of excellent quality given the resolution and anisotropy. The structure and structure factors have been deposited in the Protein Data Bank (PDB) with accession code 4CJ7 and statistics of the data and model are summarised in Table 1.

**Table 1:** Crystallographic data and refinement statistics.

Statistics	Native 1	Native 2
*Protein*	Crenactin *Pyrobaculum calidifontis* 1-432	Crenactin *Pyrobaculum calidifontis* 1-432
NCBI IDs	YP_001056518.1	YP_001056518.1
*Data collection*		
Beamline wavelengths (Å)	Diamond I04-1	Diamond I02
*Method crystal*	Native	Native
Space group	P4_1_2_1_2	P4_1_2_1_2
Cell (Å)	71.9, 71.9, 423.5	78.1, 78.1, 419.0
*Scaling*		
Resolution (Å)	3.5	3.2
Completeness (%)^a^	99.8 (99.9)	99.9 (99.9)
Multiplicity^a^	6.1 (6.6)	12.6 (12.9)
(*I*)/σ(*I*)^a^	7.8 (2.2)	8.6 (1.1)
CC1/2^a^	0.991 (0.828)	0.995 (0.818)
*R*_pim_^a^	0.101 (0.420)	0.091 (0.633)
*Refinement*		
*R*/*R*_free_^b^		0.238 (0.295)
Model		2 monomers packing as continuous filament 0 waters, 2 ADP, 0 Mg
Bond length rmsd (Å)		0.012
Bond angle rmsd (°)		1.975
Favoured (%)^c^		99.2 (748)
Disallowed (%)^c^		0.3 (2)
PDB ID		4CJ7

a Values in parentheses refer to the highest resolution bin.

b 5% of reflections were randomly selected before refinement.

c Percentage of residues in the Ramachandran plot (PROCHECK); number of residues in parentheses.

### 2.5 Polymerisation

The reaction was carried out with 0.3 mg/ml of purified *Pc* crenactin in different polymerisation buffers (50 mM ammonium hydrogen carbonate, 50 mM NaCl or 50 mM Tris/HCl, 600 mM to 2 M KCl, pH 7.4), supplemented with 2 mM ATP and 4 mM MgCl_2_ at room temperature for 30 min.

### 2.6 Negative stain electron microscopy

Freshly purified crenactin was polymerised and applied to glow-discharged EM grids covered with continuous carbon. After 30 s, excess sample was blotted away, and the grid was washed twice with 2% (w/v) uranyl acetate in water and stained for up to 30 s. When poly-l-lysine (PLL) was used to change the carbon surface of the EM grids, 1 mg/ml of low molecular weight PLL (Sigma P7890) was prepared in water and applied to a glow discharged grid for 10 min. Excess liquid was then blotted away and the grids were allowed to dry for a few minutes before sample application and negative staining as before. Samples were dried and transferred into a Tecnai T12 electron microscope (FEI Company) operated at 120 kV and imaged on a CCD camera. Averaging was performed with the EMAN2 suite [20].

### 2.7 Rotary shadowing

Samples were treated first as for negative stain EM (above). After uranyl acetate had been applied, grids were dried under high vacuum for 2 h. Rotary shadowing was performed using an Edwards E306A coating system with a platinum wire wound around a ‘V’ shaped tungsten wire at an oblique angle of 5° and with a sample-to-source distance of 10 cm.

### 2.8 Electron cryomicroscopy and image processing

Purified crenactin was polymerised and applied to glow-discharged 200-mesh R2/2 Quantifoil holey grids (Agar Scientific). The grids were blotted and frozen in liquid ethane using a Vitrobot (FEI Company). Samples were transferred to an FEI Tecnai G2 Polara electron microscope operated at 300 kV. Images were recorded on a FEI Falcon 2 4 × 4 k direct detection camera (magnification 40.3 k ×, pixel size 3.3 Å). Image processing for diffraction was performed using the MRC suite of programs [21].

## 3 Results

**Fig. 1:**
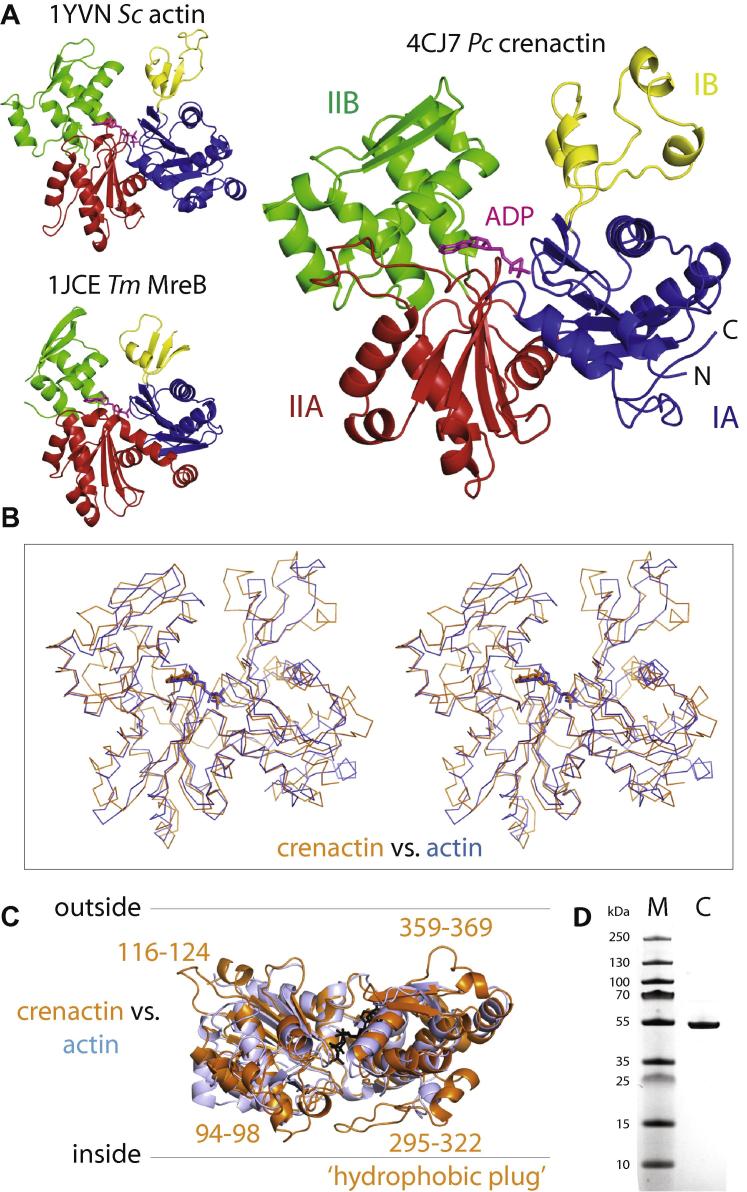
Crystal structure of *Pyrobaculum calidifontis* crenactin at 3.2 Å resolution. (A) The structure of crenactin shows the canonical domain architecture of the actin family of proteins, split into two domains with subdomains IA, B and IIA, B (right). The nucleotide (ADP) is bound in a cleft between the two domains I and II. When compared to eukaryotic and bacterial actin MreB (left) [33], crenactin is more closely related to actin. (B) Stereo view of superposition of *Pc* crenactin and eukaryotic (yeast, PDB 1YVN) actin. RMSD is 1.6 Å over 1117 atoms (1.9 Å over 332 Cα), compared to 2.2 Å with MreB (not shown). (C) When compared to eukaryotic actin, crenactin contains several larger loops on the outside and this is most pronounced for the loops between residues 295–322. In actin this is called the ‘hydrophobic plug’ since it is located on the inside of the double helical filament and makes inter-protofilament contacts holding the two strands together. (D) Coomassie-stained SDS–PAGE gel showing SUMO-cleaved and purified *Pc* crenactin as used for this study.

We produced *P. calidifontis* crenactin as a histidine-tagged SUMO fusion protein in *E. coli*. After nickel-affinity purification and SUMO protease cleavage, followed by size exclusion chromatography, the procedure yields pure and full-length protein with no residues added or removed at low yields (0.1–0.5 mg/l of culture) (Fig. 1D). The protein crystallises readily, forming long rods and needles in many conditions in our 1920-condition initial screen. The best conditions yielded crystals that belong to space group P41212 with a very long *c*-axis (Table 1) and diffract to 3.2 Å only, with the additional complication that diffraction is fairly anisotropic perpendicular to the long axis. The structure was solved by molecular replacement with eukaryotic actin (PDB code 1YVN, see Section 2 for details) and subsequent multi-crystal averaging using a second, non-isomorphous crystal in the same space group but with significantly different cell constants (Table 1). Since each asymmetric unit in the two forms Natives 1 and 2 contained two monomers, it yielded 4-fold averaging, greatly improving the very poor phases coming from the weak molecular replacement solutions. This enabled automatic model building and manual rebuilding and refinement, using 2-fold non-crystallographic symmetry, to produce a high confidence structure, refined at 3.2 Å resolution with good *R*-factors and geometry (Table 1).

As was expected, the resulting structure shows the canonical fold of actin-like proteins (Fig. 1A, right). What was not expected is the degree of similarity. When we performed database searches against all known structures in the Protein Data Bank (PDB), the first few dozen hits were all eukaryotic actins (top hit: 4M63, RMSD 1.76 Å over 325 Cα atoms [22]). A superposition of crenactin and yeast 1YVN actin yielded an RMSD of 1.62 Å over 1117 atoms (1.98 Å over 332 Cα atoms), surprisingly small values, given that the global sequence identity between crenactin and actin is only 20.3% (yeast actin PDB code 1YVN vs. crenactin; 36.3% similarity) (Fig. 1B). The low RMSD explains why the structure could be solved by molecular replacement. When compared to bacterial actin MreB, which is also present in some archaea, the similarity to crenactin is significantly lower with an RMSD of 2.2 Å over 1043 atoms (2.38 Å over 275 Cα atoms), as was expected from previous work [9] (Fig. 1A, left).

Although crenactin is very closely related to eukaryotic actin in structure, crenactin’s sequence is longer than actin’s (432 for crenactin vs. 375 for yeast actin) and the extra residues are consumed to make a number of surface loops bigger (Fig. 1C). This is most pronounced in loops 295–322, which corresponds to a loop in actin that is involved in the formation of double helical filaments and has also been termed the ‘hydrophobic plug’ [23].

**Fig. 2:**
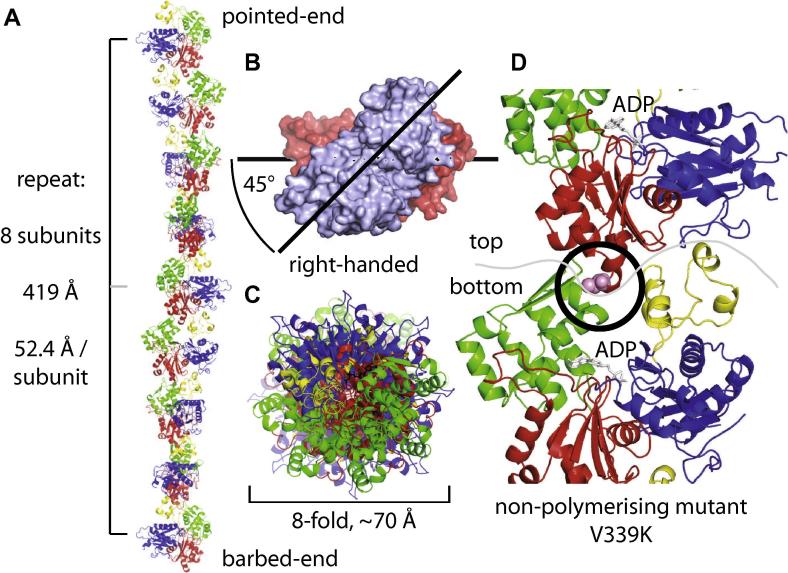
Crystals contain a steep right-handed helix with 8 subunits per repeat. (A) The crystals contain one complete turn of a crenactin protofilaments. The 360° repeat is 419 Å long and contains exactly 8 subunits at 52.4 Å repeat distance per subunit, as determined from the cell constants (Table 1). (B) Top-view of the non-crystallographic dimer (looking along the protofilament in (A) reveals the right-handed twist of the protofilaments. (C) Top-view of the entire 8-subunit protofilament repeat, revealing the overall 8-fold symmetry and the dimensions of the protofilaments in the lateral dimension with around 70 Å width. (D) Location of V339, which was mutated to K to produce the non-polymerising mutant crenactin, in a loop at the tip of subdomain IIA. In protofilaments of crenactin the tip inserts into the cleft between subdomain IB and IIB of the next subunit and presumably the mutation abrogates this interaction since V339 normally enters mainly hydrophobic surroundings upon polymerisation.

**Fig. 3:**
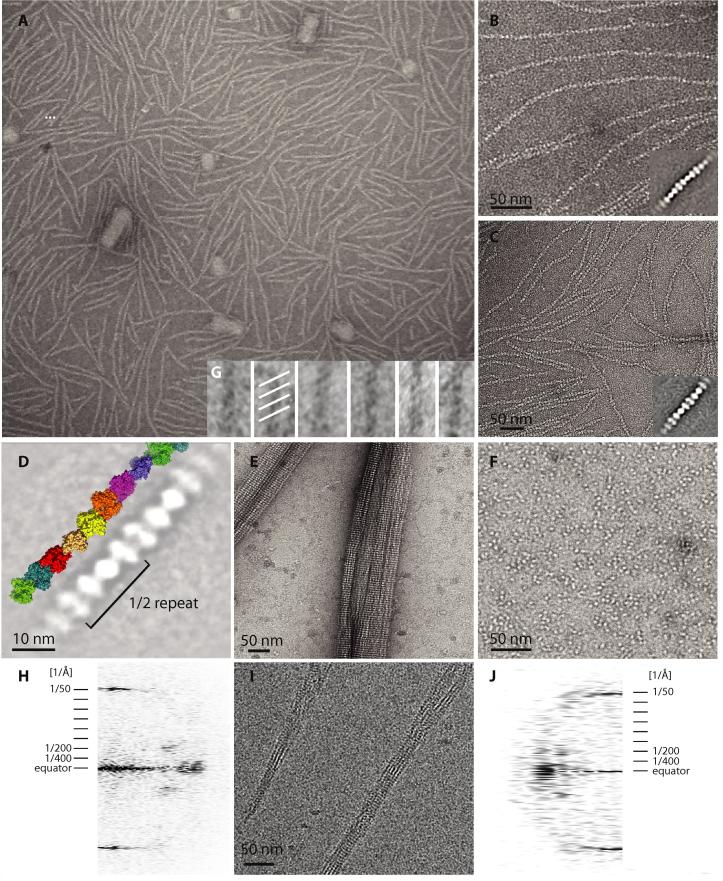
Crenactin forms helical filaments as shown by electron microscopy. (A) Field view of negatively-stained *Pc* crenactin filaments, formed on grids treated with poly-l-lysine, revealing a well-behaved sample. (B) Close up of the same poly-l-lysine condition and single-particle average (1532 particles). (C) Close-up of crenactin filaments polymerised in the presence of 600 mM KCl and single particle average (622 particles). (D) Comparing the single-particle averages of crenactin filaments with the protofilament present in the crystals reveals close similarity. Given the resolution obtained it is currently not possible to discern whether the filaments in EM are single protofilaments or actin-like double helical. (E) Crenactin bundles formed by 2 M KCl concentrations. (F) Demonstration that crenactin carrying the mutation V339K does not form filaments. (G) Inset at bottom of panel B: rotary shadowing after negative staining shows the filaments to be right-handed, confirming (together with panel D) that the helical parameters of the filaments in the crystals are identical to the ones observed here by EM. (H) Computed diffraction image of negatively stained crenactin filaments (similar to the ones in panel E). The first layer line appears at around 1/200 Å^−1^. This probably indicates that the filaments have the same 45° twist per subunit as in the crystals, but additional symmetry from the double helical filament halves the repeat length by non-staggered subunits, as indicated by the bead-like appearance of the filaments, for example in panels B and C. (I) Electron cryomicroscopy of crenactin shows bundles. (J) Computed diffraction image of crenactin filaments, imaged by electron cryomicroscopy, showing the same 1/200 Å^−1^ repeat length as in negative stained crenactin bundles.

The crystals of crenactin have a very long c axis because they contain essentially infinite helical protofilaments (Fig. 2A). Eight subunits form a complete turn of 360° over a distance of 419 Å, the length of the unit cell of the crystals. This means that each subunit repeat is 52.4 Å and the angle from subunit to subunit is 45°. The helix is right-handed, as for actin filaments (Fig. 2B). The crenactin crystals contain a protofilament, made out of a single strand of crenactin subunits and this leads to an overall protofilament thickness of only 70 Å (Fig. 2C). Actin, of course, forms double helical filaments made out of two protofilaments, arranged in a parallel fashion with the same surfaces facing inwards (Fig. 1C, inside) [23], [24]. To our knowledge, this is the first instance of an actin-like protein forming a complete helical protofilament in crystals. For actin, tricks had to be used to crystallise fragments or inhibited complexes [22] and for ParM (and actin) helical reconstruction electron cryomicroscopy was employed to obtain structures and these are still at somewhat lower resolutions [25]. To test the validity of the helical arrangement as we observed it in the crystals we designed a non-polymerising mutant of crenactin protein based on the longitudinal interface formed when crenactin subunits come together for the protofilament (Fig. 2D). We selected V339K, which sits at the top of subdomain IIA. This region inserts into the cleft between subdomain IB and IIB of the lower subunit (Fig. 2D) and V339 then becomes part of a hydrophobic environment, hence the change to lysine in an attempt to disrupt the ability to form this interface and therefore polymerisation. A similar approach worked very well for ParM, although L163 is located in a neighbouring turn [25]. The V339K mutation abrogated filament formation, as was intended and deduced from its location in the longitudinal protofilament interface between two subunits (Figs. 2D and 3F).

We then investigated what filaments crenactin formed using negative staining electron microscopy (Fig. 3). Surprisingly, crenactin did not show any filaments when ATP was added. When the carbon-coated grids were treated with poly-l-lysine, however, very uniform filament formation could be observed (Fig. 3A and B). The filaments consisted of several dozen subunits in length and are well resolved so that single particle averaging could be performed (Fig. 3B and C, insets and D). Similarly, when high salt was used (from 600 mM to 1.2 M KCl, no poly-l-lysine added), filament formation could be induced to equivalent levels of efficiency (Fig. 3C) and the average of these filaments showed them to be identical to the ones formed on the poly-l-lysine-treated grids. When even higher salt concentrations were used (2 M KCl), crenactin formed large bundles, presumably because of a further increase in hydrophobicity and increased attraction between the individual filaments, competing with filament–carbon interactions (Fig. 3E).

We then compared the averages of the filaments as observed in EM with the protofilaments present in the crenactin crystals (Fig. 3D). It became immediately obvious that the two are very similar: they repeat every 8 subunits, have comparable thicknesses and are similar in subunit appearance, possibly showing the two-domain architecture of crenactin (and indeed all actin-like proteins) as two blobs (see Section 4). We then determined the handedness of the filaments using electron microscopy by rotary shadowing the negatively stained filaments. This images the top of the helices only, and the direction of stripes indicates the handedness; striations going upwards mean right handed, and this is what we observed (Fig. 3G). It is the same handedness as the protofilaments in the crenactin crystals so we conclude that the protofilaments in the crystals are very similar in helical parameters to the ones we investigated by electron microscopy.

## 4 Discussion

We have demonstrated that crenactin’s three-dimensional structure is surprisingly closely related to actin and that it forms very similar tightly wound helical filaments with 8 subunits per complete turn, both in the crystals and when investigated by electron microscopy.

It is not clear, however, if the filaments we observed by EM (Fig. 3) are single protofilaments as observed in the crystals (Fig. 2). Single protofilaments, if they exist at all in solution, should be quite fragile and long single filaments of any actin-like protein have not been reported to our knowledge. ParM forms parallel left handed double helical filaments [26] and MreB forms antiparallel straight double filaments [6], while actin forms right-handed double helical filaments [23]. For this issue to become resolved we will have to obtain high-resolution electron cryomicroscopy data on crenactin filaments and possibly even a high-resolution helical reconstruction. Our low resolution computed diffraction images of crenactin bundles as observed by negative stain and electron cryomicroscopy (Fig. 3H and J) show layer lines at ∼1/200 Å^−1^. It unlikely that this is the complete repeat per protofilament, since the angle from subunit to subunit would then have to be 90°, unrealistically large. If we assume a double filament, a layer line would appear at 1/200 Å^−1^ only if the two strands are not staggered, producing a complete repeat after only 180° rotation, half of the true 360° complete turn because of additional 2-fold symmetry in the filament. This is supported by the appearance of the filaments in the electron microscopy images, where they look like beads on a string, which would not be expected if subunits were staggered in a double filament (as, for example, for actin and ParM).

In this context it is worth mentioning again that crenactin’s hydrophobic plug is much larger (loops 295–322, Fig. 1C). When superimposed on canonical double helical F-actin filament structures, crenactin’s hydrophobic plug creates major clashes on the inside of the filament where the two protofilaments of actin interact to form the double helix. This may explain the occurrence of single protofilaments of crenactin under our experimental conditions or, more likely, might indicate that the double filament is not staggered as for actin and the hydrophobic plug loop binds to an entirely different surface on another subunit.

Our work demonstrates the strong structural similarity between crenactin and actin. They are so closely related that one needs to contemplate two possible evolutionary scenarios: crenactin may be the result of horizontal gene transfer between an eukaryote and Crenarchaeum (or more precisely one of its progenitors). Or, crenactin is the primordial actin. While these things can never be determined with complete certainty, it seems more likely to us that crenactin has not been horizontally transferred. We proposed horizontal transfer be the most likely case for BtubAB, a very close homologue of eukaryotic tubulin in some *Prosthecobacter* species [27], [28]. In that case, sequence conservation is much higher and BtubAB proteins are much more closely related to tubulins than anything else in the genome is related to eukaryotic homologues, so that horizontal gene transfer seems the most likely scenario for BtubAB. For crenactin, sequence conservation is low (20% identity to eukaryotic actin) and this is well in line with many other proteins in Crenarchaea. Another example of horizontal transfer, in this case of actin and profilin, has been documented for *Microcystis*, a cyanobacterium [29]. Again, these genes are much more ‘eukaryotic’ than anything else in the genome of these organisms, making it possible to differentiate horizontal from vertical/lineage transfer.

Crenactin being a (or the) primordial actin supports the idea that Crenarchaea (or more precisely one of their progenitors) developed into the eukaryotic lineage, after bacterial endosymbionts were included in several independent events. This is supported by widespread similarities within information processing machinery [10]. And recently it has been demonstrated that some Crenarchaea use the ESCRT system for cytokinesis [30], [31], making a possible evolutionary connection with the late stages of eukaryotic cytokinesis, that also involve ESCRT III [32].
